# The Synergistic Reducing Drug Resistance Effect of Cisplatin and Ursolic Acid on Osteosarcoma through a Multistep Mechanism Involving Ferritinophagy

**DOI:** 10.1155/2021/5192271

**Published:** 2021-12-21

**Authors:** Zhen Tang, Hui Dong, Tian Li, Ning Wang, Xinghui Wei, Hao Wu, Yichao Liu, Wei Wang, Zheng Guo, Xin Xiao

**Affiliations:** ^1^Department of Orthopedics, Xijing Hospital, Fourth Military Medical University, Xi'an, China; ^2^School of Basic Medicine, Fourth Military Medical University, Xi'an, China; ^3^Department of Immunology, State Key Laboratory of Cancer Biology, Fourth Military Medical University, Xi'an, China; ^4^Department of Orthopaedics, Tangdu Hospital, Fourth Military Medical University, Xi'an, China

## Abstract

Increasing evidence suggests that traditional Chinese medicine strategies are obviously beneficial for cancer treatment, but scientific research on the underlying molecular mechanisms is lacking. We report that ursolic acid, a bioactive ingredient isolated from Radix Actinidiae chinensis, has strong antitumour effects on osteosarcoma cells. Functional studies showed that ursolic acid inhibited tumour cell proliferation and promoted the apoptosis of a variety of osteosarcoma cells. Ursolic acid had a synergistic cytotoxic effect with cisplatin on osteosarcoma cells. In a mouse osteosarcoma xenograft model, low-dose cisplatin combined with ursolic acid significantly reduced tumour growth. Notably, ursolic acid reversed weight loss in mice treated with cisplatin. Mechanistic studies showed that ursolic acid degraded ferritin by activating autophagy and induced intracellular overload of ferrous ions, leading to ferroptosis. In addition, ursolic acid enhanced the DNA-damaging effect of cisplatin on osteosarcoma cells. Taken together, these findings suggest that ursolic acid is a nontoxic adjuvant that may enhance the effectiveness of chemotherapy in osteosarcoma.

## 1. Introduction

Osteosarcoma (OS) is a type of sarcoma that originates from osteoblast mesenchymal cells and is most commonly seen in children and adolescents aged 15-19 years [1]. Current treatment strategies, including chemotherapy combined with aggressive surgical resection, have significantly improved the survival of patients with OS [2]. However, the recurrence rate is still 30-40%, and the 10-year survival rate is 20-30% due to pulmonary metastasis [3]. Chemotherapy, such as cisplatin (Cis), is the most effective treatment for cancer and OS [4–6]. However, these chemotherapeutic agents are limited by the acquisition of resistance and serious side effects at high doses. Therefore, there is an urgent need to investigate alternative anticancer approaches, such as the use of food and herbal supplements in combination with current chemotherapy to improve the therapeutic effectiveness of existing drugs. The development of effective, nontoxic therapeutic strategies, i.e., the application of natural active substances with proven anticancer effects, may be a more promising approach for the prevention and treatment of OS.

Evidence-based studies on traditional Chinese medicine (TCM) strategies for cancer treatment have shown that TCM compounds target tumour cells and treat the whole body. Bioactive TCM compounds exert antitumour effects through multilevel targeting and affecting signalling pathways [7]. Therefore, TCM compounds can be used as adjunctive treatments for cancer. Ursolic acid (UA) is a natural pentacyclic triterpenoid carboxylic acid found in Radix Actinidiae chinensis. Studies have shown that UA has a wide range of biological activities, including antiviral, antibacterial, hepatoprotective, and anti-inflammatory effects [8]. In addition, it is involved in the prevention of and protection against cancer. Its antitumour effect is due to its ability to prevent tumour occurrence, inhibit proliferation, and induce apoptosis. Previous studies have shown that UA can inhibit the proliferation and induce apoptosis of prostate, lung, pancreatic, and other tumour cells [9]. In addition, UA has been reported to inhibit tumour progression, induce tumour cell differentiation, and inhibit angiogenic activity [10]. UA has also been found to exert chemoprophylaxis effects in different animal models by inhibiting tumour invasion. However, the antitumour effects of UA, its effectiveness as an adjuvant chemotherapy in OS cells, and its underlying molecular mechanism remain unclear.

In this study, the effects of UA components from Radix Actinidiae chinensis on OS cells were comprehensively studied. We used two OS cell lines and a mouse tumour xenograft model to demonstrate that UA is nontoxic and has strong antitumour activity and a marked effect against chemotherapy resistance in OS cells in vitro and in vivo when combined with Cis. These findings suggest that UA may serve as an adjuvant to enhance the therapeutic effectiveness of current chemotherapy-based regimens.

## 2. Materials and Methods

### 2.1. Cell Culture and Reagents

The human OS cell lines HOS and 143B were purchased from the Cell Bank of the Chinese Academy of Medical Science (Shanghai, China). HOS and 143B cells were grown in Eagle's minimum essential medium (Gibco, Los Angeles, CA) containing 10% foetal bovine serum (Gibco), 100 U/mL penicillin, and 100 *μ*g/mL streptomycin at 37°C and 5% CO_2_. Cis, UA, desferrioxamine (DFO), and 3-methyladenine (3-MA) were purchased from Sigma-Aldrich (St. Louis, Missouri, USA).

### 2.2. Cell Viability Assay

Adhering to the previous protocol [11], cells were seeded in a 96-well plate at a density of 1 × 10^4^ cells per well in 100 *μ*L medium overnight. After the cells were treated with reagents and incubated for 24 h, 10 *μ*L Cell Counting Kit-8 (CCK-8) reagent was added according to the manufacturer's instructions. The absorbance was measured at 450 nm with a microplate reader.

### 2.3. Clonogenic Assay

HOS and 143B cells in the logarithmic growth phase were seeded into a 10 cm dish at a density of 200 cells per millilitre. After treatment with Cis (20 *μ*mol/L), UA (35 *μ*mol/L), or Cis (20 *μ*mol/L)+UA (35 *μ*mol/L) for 2 weeks, cell clones were fixed with 4% paraformaldehyde for 15 min. The cell clones were stained with crystal violet solution at room temperature for 15 min before they were photographed.

### 2.4. Cell Apoptosis Analysis

To study the synergistic effect of Cis and UA on apoptosis, HOS and 143B cells were incubated with Cis (20 *μ*mol/L) or UA (35 *μ*mol/L) for 24 h and then harvested and stained using an Annexin V-PE Apoptosis Detection Kit (BD, Shanghai, China). The number of apoptotic cells was quantified by flow cytometry. All experiments were performed in triplicate.

### 2.5. Live/Dead Staining Assay

HOS and 143B cells were seeded in 24-well plates (2.5 × 10^4^/well) and cultured overnight. Cis (20 *μ*mol/L), UA (35 *μ*mol/L), or Cis (20 *μ*mol/L)+UA (35 *μ*mol/L) was added to the wells for 24 h. Then, a calcein-AM/PI staining kit (Beyotime, Shanghai, China) was used to detect dead and live cells. PI was used to stain dead cells, and calcein-AM was used to stain live cells.

### 2.6. 5-Ethynyl-2′-deoxyuridine Assay (EdU) Incorporation Assay

HOS cells were seeded in 24-well plates and cultured overnight in complete medium. Cis (20 *μ*mol/L), UA (35 *μ*mol/L), or Cis (20 *μ*mol/L)+UA (35 *μ*mol/L) was added to the wells. Twenty-four hours after treatment, cell proliferation was measured with the EdU Cell Proliferation Assay Kit (Beyotime, Shanghai, China) according to the manufacturer's instructions. The cell nuclei were stained with 1 *μ*g/mL DAPI (Solarbio, Beijing, China) for 5 min. The proportion of EdU-incorporated cells was determined by fluorescence microscopy.

### 2.7. Cell Cycle Analysis

HOS cells were seeded in 6-well plates and treated with Cis (20 *μ*mol/L), UA (35 *μ*mol/L), or Cis (20 *μ*mol/L)+UA (35 *μ*mol/L) for approximately 24 h. Then, the cells were collected and fixed in ice-cold 70% ethanol at 4°C overnight. A Cell Cycle Staining Kit (MultiSciences, Hangzhou, China) was used to analyse the cell cycle phases according to the manufacturer's protocol. After being washed three times with cold PBS, the cells were immersed in RNase A for 30 min at room temperature and then incubated with propidium iodide (PI) in the dark. The samples were analysed by flow cytometry.

### 2.8. Determination of Reactive Oxygen Species (ROS) Generation

HOS cells (5 × 10^5^) plated in six-well plates were incubated with Cis (20 *μ*mol/L), UA (35 *μ*mol/L), or Cis (20 *μ*mol/L)+UA (35 *μ*mol/L) for approximately 24 h, and then, the medium was replaced with 1 mL fresh medium containing 5 *μ*M BODIPY 581/591 C11 (Glpbio, Guangzhou, China) for 30 min at 37°C. The cells were then washed twice and analysed under a fluorescence microscope.

Cellular ROS levels and superoxide levels were measured using 2′,7′-dichlorofluorescein diacetate (DCF-DA). According to the manufacturer's protocol, HOS and 143B cells were washed with PBS and stained with DCF-DA (4 *μ*M) in the dark at 37°C for 30 min. Flow cytometry was used to determine ROS production.

### 2.9. Detection of Malondialdehyde (MDA) and 4-Hydroxynonenal (4-HNE)

The Lipid Peroxidation (MDA) Assay Kit (MAK085, Sigma-Aldrich, USA) and Lipid Peroxidation (4-HNE) Assay Kit (ab238538, Abcam, USA) were used to measure the concentration of MDA and 4-HNE in cell lysates according to the manufacturer's instructions. For MDA detection, the samples were mixed with thiobarbituric acid and incubated at 100°C for 1 h. The MDA concentration was determined at 532 nm absorbance. For 4-HNE detection, the samples were added into the 4-HNE conjugate-coated plate and incubated for 10 min. Then, the conjugated 4-HNE was incubated with anti-4-HNE antibody and secondary antibody-HRP. Finally, substrate solution was added, and the absorbance at 450 nm was measured. Herein, the amount of MDA or 4-HNE was calculated in 1 × 10^6^ cells after treatment.

### 2.10. Measurement of Intracellular Fe^2+^ Content

FerroOrange (Mkbio, Shanghai, China) was used to measure intracellular and mitochondrial Fe^2+^ levels according to the manufacturer's protocol. HOS cells were treated with Cis, UA, UA/Cis, UA/Cis/DFO, or UA/Cis/3-MA and stained with 1 *μ*M FerroOrange for 30 min at 37°C. Images were acquired using a fluorescence microscope.

### 2.11. Autophagic Flux Analysis

mCherry-GFP-labelled LC3 was used to analyse autophagic flux in cells according to the manufacturer's instructions. HOS cells were transfected with mCherry-GFP-LC3 adenovirus for 6 h and then incubated with Cis (20 *μ*mol/L), UA (35 *μ*mol/L), or Cis (20 *μ*mol/L)+UA (35 *μ*mol/L) for 24 h. mCherry-GFP-LC3 expression was tracked and imaged by fluorescence microscopy.

### 2.12. Western Blotting

OS cells were lysed with RIPA buffer and centrifuged at 12000 rpm for 15 min to collect the supernatant. The protein samples were separated by 10% SDS-PAGE and transferred to PVDF membranes (Millipore, Billerica, MA, USA). The PVDF membranes were blocked with 5% skim milk for 1.5 h and then incubated overnight with primary antibody at 4°C. After being washed with TBST 3 times (15 min each), the membranes were incubated with an HRP-conjugated secondary antibody at room temperature for 1 h. The protein bands were visualized with an Amersham Imager 600. Primary antibodies against Beclin-1 (ab207612, Abcam), LC3 (ab192890, Abcam), P62 (ab109012, Abcam), *γ*-H2AX (ab81299, Abcam), GPX4 (DF6701, Affinity), TFR (AF5343, Affinity), and NCOA4 (DF4255, Affinity) were diluted 1 : 1000; an antibody against GAPDH (#5174, CST, USA) was diluted 1 : 2000.

### 2.13. Transmission Electron Microscopy

Cells cultured in a 6-well plate were treated with drugs for 24 h and fixed with 2.5% glutaraldehyde in PBS. After being washed in 0.1 M PBS, the cells were treated with 0.1% Millipore-filtered cacodylate-buffered tannic acid and postfixed with 1% osmium tetroxide at 4°C for 30 min. Then, the cells were incubated in a graded series of acetone solutions for 10 min each. After dehydration and embedding for 2 h at 60°C, the cells were embedded, and the embedded tissues were sliced at a thickness of 50~70 nm for follow-up observation using a transmission electron microscope.

### 2.14. Immunofluorescence

OS cells were seeded in 24-well plates at a density of 5 × 10^4^ cells per well and treated with the indicated drugs for 24 h. Then, the cells were fixed in 4% paraformaldehyde for 20 min and incubated with a primary antibody against LC3 (ab192890, Abcam), *γ*-H2AX (ab81299, Abcam), and TFR (AF5343, Affinity) at 4°C overnight. The cells were washed with PBS three times for 3 min each and then incubated with secondary antibodies for 1 h at room temperature. DAPI was used to stain the nuclei for 1 min. The samples were sealed and observed under a fluorescence microscope.

### 2.15. Xenograft Model of Human OS

All animal experiments were carried out following the guidelines of the Ethics Committee of the Fourth Military Medical University (IACUC No. 2020-1004) and were performed according to institutional guidelines for the care and use of laboratory animals. The guidelines were complied with the *Guide for the Care and Use of Laboratory Animals* NIH publication No. 86-23, revised 2011. NU/NU mice (the Fourth Military Medical University, Shaanxi, China) were injected with 143B cells (100 *μ*L, 5 × 10^7^ cells/mL, i.h.). Seven days after the injection, the mice were divided into 6 different groups (*n* = 3) and intraperitoneally injected with different drugs twice a week. Then, on day 28, the mice were sacrificed, and the tumours in the different groups were weighed. Body weight and tumour size were measured every 3 days from day 7 to day 28. The tumour tissue was fixed with 4% paraformaldehyde, embedded in paraffin, and cut into 4 *μ*m thick sections for haematoxylin-eosin (H&E) and immunofluorescence staining.

### 2.16. Statistical Analysis

Statistical analyses were performed with GraphPad Prism 8.2 (GraphPad Software, Inc., La Jolla, CA, USA). The data are presented as the mean ± SD of 3 independent experiments. Statistical significance between the groups was analysed with one-way analysis of variance (ANOVA). *p* < 0.05 was considered statistically significant.

## 3. Results

### 3.1. Synergistic Antitumour Effect of UA and Cis on OS Cells In Vitro

**Figure 1:**
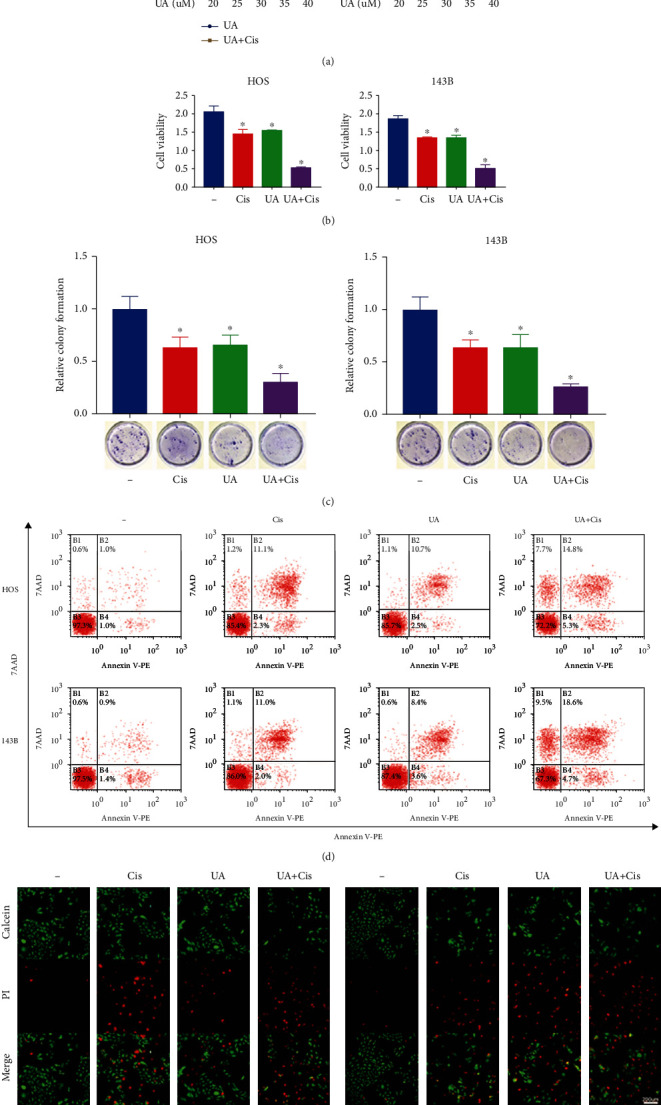
Synergistic antitumour effect of UA and Cis on OS cells in vitro. HOS and 143B cells were treated with different concentrations of UA and Cis for 24 h, and cell viability was assessed by the CCK-8 assay (a) (*n* = 3). The CCK-8 assay was used to evaluate cell viability under UA and/or Cis treatment (b) (*n* = 3). The colony formation ability of HOS and 143B cells was assessed after monotherapy or treatment with the combination of UA and Cis for 14 days (c) (*n* = 3). HOS and 143B cells were treated with UA and/or Cis for 24 h, and the apoptosis rate was measured by flow cytometry (d) (*n* = 3). HOS and 143B cells were pretreated with UA and/or Cis for 24 h and subjected to calcein-AM/PI staining (e) (*n* = 3, scale bar: 200 *μ*m; calcein-AM: live cells; PI: dead cells). The data are presented as the mean ± SD. ^∗^*p* < 0.05 vs. the control group.

To determine the optimal concentration of UA for treating OS cells, we used the CCK-8 assay to determine the effects of different concentrations of UA on the proliferation of HOS and 143B cells. The results showed that 35 *μ*M UA combined with 20 *μ*M Cis had the most obvious effect on the proliferation of OS cells (Figure 1(a)). Then, the CCK-8 assay was used to evaluate the effects of Cis and UA and their combination on the proliferation of HOS and 143B cells. Cis or UA treatment significantly decreased cell viability, and this decrease was more significant in the combined UA and Cis treatment group (Figure 1(b)). The results of the clonogenic assay also showed that OS cell viability was the lowest in the UA+Cis group, followed by the monotherapy group (Figure 1(c)). Flow cytometry was used to analyse the apoptosis of HOS and 143B cells after various drug treatments. The results showed that the apoptosis rate of OS cells was the highest in the UA+Cis group and was higher in the UA+Cis group than that in the Cis or UA alone group but that the apoptosis rate in all three groups was significantly higher than that in the control group (Figure 1(d)). We performed calcein-AM/PI staining to further clarify the synergetic effect of UA and Cis on HOS and 143B cells. The experiment demonstrated that the combination of UA and Cis synergistically increased the number of dead cells and decreased the number of viable cells (Figure 1(e)).

### 3.2. The Combination of UA and Cis Inhibits Proliferation and Induces DNA Damage in OS Cells

**Figure 2:**
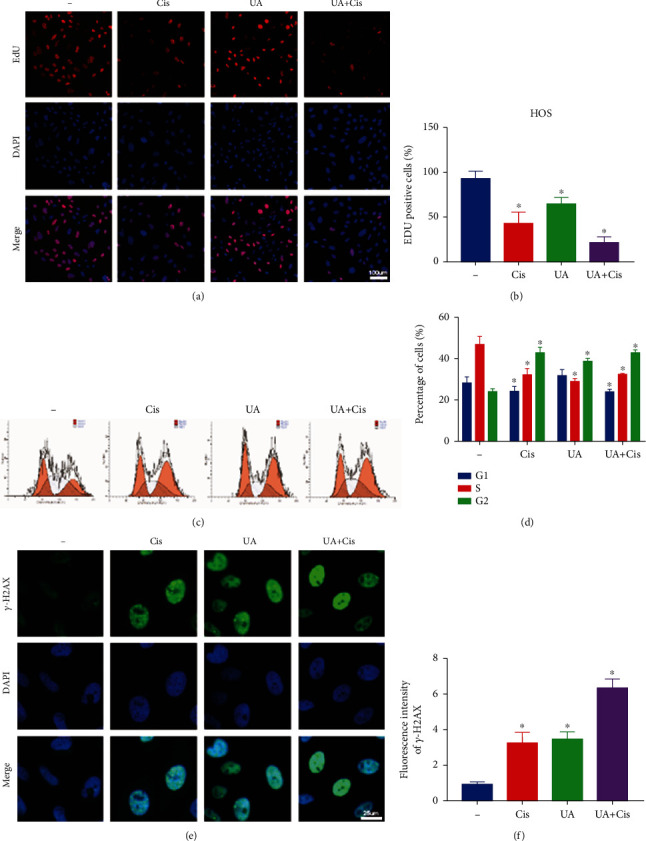
Combination treatment with UA and Cis inhibited OS cell proliferation by increasing DNA damage in OS cells. The proliferation of OS cells was assessed by the EdU incorporation assay after monotherapy or treatment with the combination of UA and Cis for 24 h (a, b) (*n* = 3, scale bar: 100 *μ*m). OS cells were treated with the combination of UA and Cis or either drug alone for 24 h, and cell cycle distribution was analysed by flow cytometry (c, d) (*n* = 3). OS cells were treated with the combination of UA and Cis or either drug alone for 24 h and stained with a *γ*-H2AX antibody (e, f) (*n* = 3, scale bar: 25 *μ*m). The data are presented as the mean ± SD. ^∗^*p* < 0.05 vs. the control group.

Subsequently, we explored whether the combination of UA and Cis can inhibit the proliferation of OS cells. As expected, both UA and Cis treatment modestly reduced the percentage of EdU^+^ cells, and UA+Cis treatment significantly reduced the percentage of EdU^+^ cells, suggesting that UA combined with Cis significantly blocked the growth of OS cells (Figures 2(a) and 2(b)). To further investigate the mechanism underlying the inhibitory effect of the drugs on OS cell growth, flow cytometry was used to analyse cell cycle distribution. As shown in Figures 2(c) and 2(d), the cell cycle distribution of HOS cells changed significantly under UA and Cis treatment, as the proportion of cells in G2 phase was significantly increased and that of cells in G1 phase was significantly decreased. Previous studies have reported that the main anticancer mechanism of Cis involves the formation of intrachain crosslinking adducts through the interaction of Cis with DNA, leading to an increase in DNA damage and cell cycle arrest [12]. Immunofluorescence was performed to assess the expression of *γ*-H2AX, a marker of early DNA damage. The results showed that UA increased DNA damage in HOS cells, while the combination therapy further significantly increased the DNA damage, as indicated by a significant increase in the fluorescence intensity of *γ*-H2AX (Figures 2(e) and 2(f)). Therefore, our experimental results suggest that UA and Cis exert a synergistic effect to inhibit OS cell proliferation and induce DNA damage.

### 3.3. Combined Treatment with UA and Cis Synergistically Increases Ferroptosis of OS Cells

**Figure 3:**
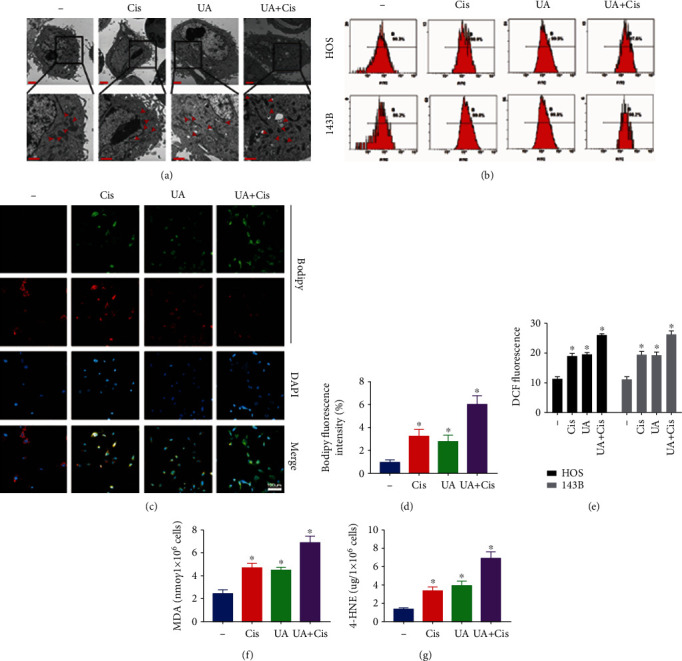
The combination of UA and Cis synergistically triggered an evident increase in cellular ROS production and lipid peroxide generation and morphological changes in mitochondria. The subcellular structural changes in HOS cells treated with the combination of UA and Cis or either drug alone for 24 h were analysed by TEM. The red arrows indicated mitochondria (a) (*n* = 3, scale bar: 2 *μ*m, scale bar: 1 *μ*m). Cellular ROS levels were measured by flow cytometry (b, e) (*n* = 3). Representative immunofluorescence images of BODIPY were obtained after UA and/or Cis treatment (c, d) (*n* = 3, scale bar: 100 *μ*m). MDA and 4-HNE levels in HOS cells treated with UA and/or Cis for 24 h (f, g) (*n* = 3). The data are presented as the mean ± SD. ^∗^*p* < 0.05 vs. the control group.

We tested whether UA combined with Cis amplifies mitochondrial dysfunction in OS cells. We focused first on mitochondrial morphology, which is upstream of mitochondrial dysfunction. Electron microscopy analysis showed that compared to monotherapy, UA+Cis treatment induced more severe changes in mitochondrial morphology, such as loss of the mitochondrial crest and an increase in the mitochondrial membrane density (Figure 3(a)). After UA+Cis administration, the mitochondria became disordered and smaller, and the network around the perinuclear region collapsed (Figure 3(a)). The formation of lipid peroxides and the accumulation of free iron are two main characteristics of ferroptosis. Therefore, oxidative stress damage was quantified by measuring (i) total ROS production in the cells with DCF-DA (Figures 3(b) and 3(e)), (ii) lipid ROS levels with BODIPY C11 (Figures 3(c) and 3(d)), and (iii) the content of secondary products of lipid peroxidation (as determined by immunofluorescence staining for MDA and 4-HNE) (Figures 3(f) and 3(g)). These results showed that ROS production and lipid peroxidation production were significantly increased by the combination treatment.

### 3.4. The Combined Use of UA and Cis Results in Iron Homeostasis Disruption in OS Cells

**Figure 4:**
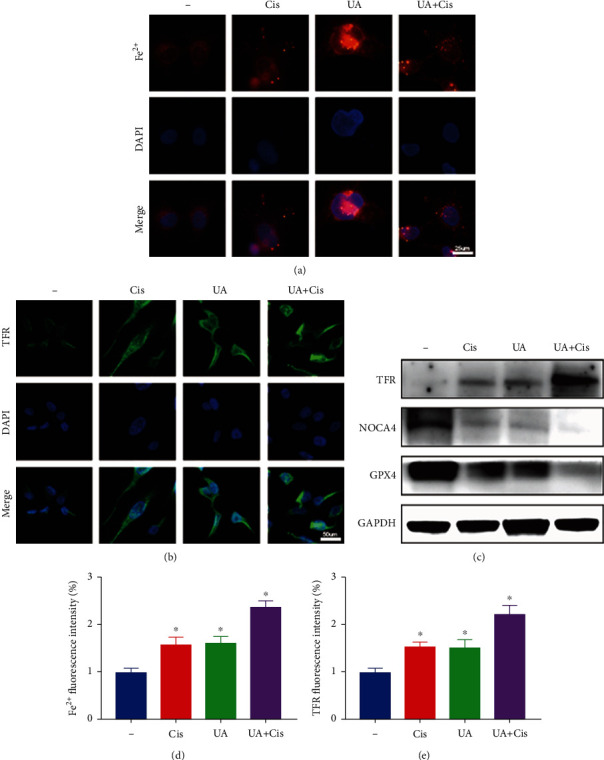
Iron accumulation in OS cells treated with the combination treatment of UA and Cis. Intracellular Fe^2+^ levels were measured after treatment with UA and/or Cis as indicated (a, d) (*n* = 3, scale bar: 25 *μ*m). The expression quantity of TFR was assessed by immunofluorescence staining of HOS cells after the indicated treatment (b, e) (*n* = 3, scale bar: 50 *μ*m). The expression of iron metabolism-related proteins in UA- and/or Cis-treated HOS cells was determined by western blotting (c) (*n* = 3). The data are presented as the mean ± SD. ^∗^*p* < 0.05 vs. the control group.

To examine whether ferroptosis is caused by an increase in free iron pools, we examined the effects of UA and Cis on iron metabolism in OS cells. We first used a selective fluorescent ferrous ion probe to monitor intracellular free iron levels. Fluorescence staining showed that UA combined with Cis resulted in significant accumulation of unstable iron, as shown by an increase in the fluorescence intensity of Fe^2+^ (Figures 4(a) and 4(d)). Next, the expression of transferrin receptor (TFR) was assessed by immunofluorescence staining (Figures 4(b) and 4(e)). Consistent with the above results, the accumulation of TFR and reduced expression of nuclear receptor coactivator 4 (NCOA4), which mediates autophagy-mediated degradation of ferritin, led to the accumulation of a large amount of nonchelated iron under UA and Cis treatment. We further investigated the expression of key proteins associated with iron homeostasis in OS cells. The results showed that the expression of proteins related to iron metabolism, such as TFR and NCOA4, was regulated by UA and that the iron starvation effect of OS cells was further enhanced under the combined action of Cis and UA. In addition, UA and Cis synergically induced the degradation of GPX4 and NCOA4, autophagy cargo receptors that bind ferritin to degrade and release free iron (Figure 4(c)). Overall, our results suggest that the combination therapy caused significant accumulation of intracellular free iron, leading to ferroptosis.

### 3.5. The Combination of UA and Cis Induces Autophagy in OS Cells

**Figure 5:**
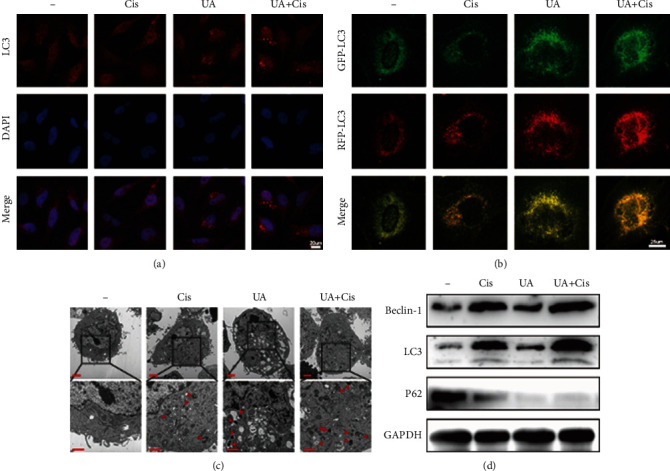
The combination of UA and Cis induced ferroptosis dependent on ferritinophagy. After treatment with UA and/or Cis for 24 h, LC3 puncta formation was analysed by immunofluorescence (a) (*n* = 3, scale bar: 20 *μ*m). Autophagic flux in 143B cells transiently expressing GFP-RFP-LC3 was monitored by fluorescence microscopy (b) (*n* = 3, scale bar: 25 *μ*m). Intracellular autophagosomes were observed by TEM (c) (*n* = 3, scale bar: 2 *μ*m, scale bar: 1 *μ*m). 143B cells were subjected to western blotting to measure the expression of LC3, P62, and Beclin-1 (d) (*n* = 3). The data are presented as the mean ± SD. ^∗^*p* < 0.05 vs. the control group.

As shown by the immunofluorescence results presented in Figure 5(a), after treatment with UA+Cis, the recruitment of LC3 to the autophagic membrane was significantly increased. Similarly, previous studies have reported that the main anticancer mechanism of Cis involves the formation of intrachain crosslinking adducts through the interaction of Cis with DNA (Figure 5(b)). Ultrastructural analysis of autophagosomes using TEM also showed that the number of intracellular autophagosomes was significantly increased after treatment with UA+Cis (Figure 5(c)). The expression levels of autophagy-related proteins were analysed by western blotting. LC3 and Beclin-1 expression was significantly increased in the UA+Cis group compared to the control group and the monotherapy group, whereas P62 expression showed a downward trend (Figure 5(d)). These results suggested that autophagy played an important role in the treatment of OS cells.

### 3.6. Pharmacologic Elimination of Free Iron and Inhibition of Autophagy Attenuate Ferroptosis Induced by UA and Cis

**Figure 6:**
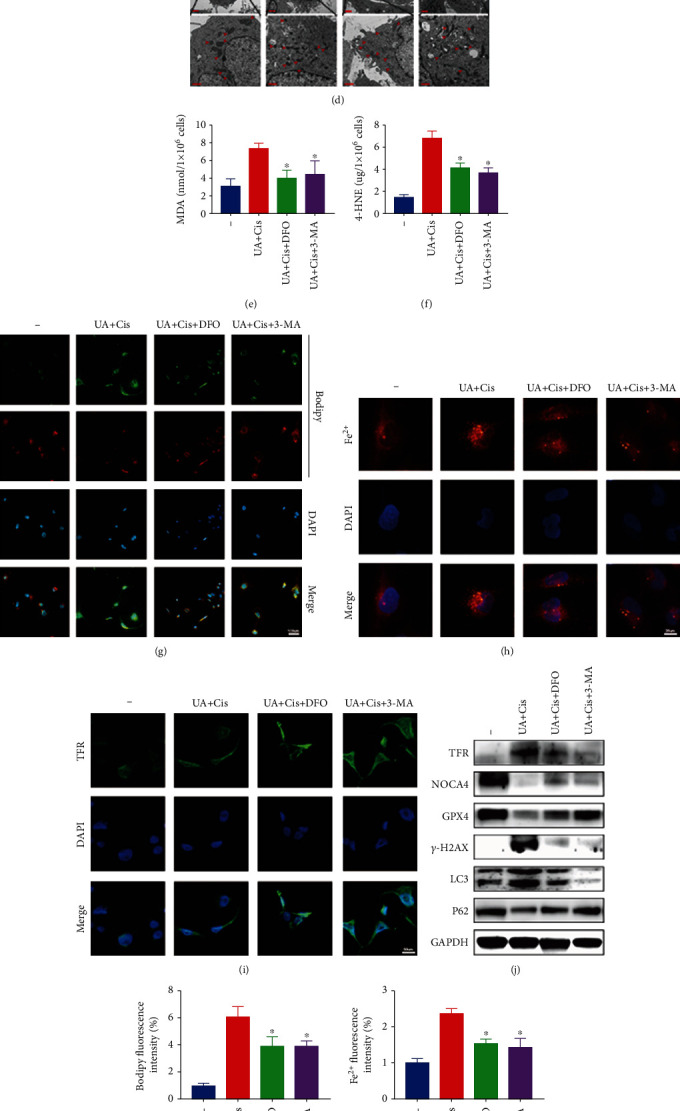
Depletion of free iron reservoirs by DFO and inhibition of autophagy by 3-MA attenuated UA/Cis-induced ferroptosis. The apoptosis rate of HOS cells treated with UA/Cis, UA/Cis/DFO, or UA/Cis/3-MA was measured by flow cytometry (a, b) (*n* = 3). The viability of HOS cells treated with Cis, UA, UA/Cis, UA/Cis/DFO, or UA/Cis/3-MA was assessed by the CCK-8 assay (c) (*n* = 3). Mitochondrial morphology changes and autophagy in HOS cells were observed by TEM (d) (*n* = 3, scale bar: 2 *μ*m, scale bar: 1 *μ*m). MDA and 4-HNE levels in HOS cells treated with UA/Cis, UA/Cis/DFO, or UA/Cis/3-MA (e, f) (*n* = 3). Representative immunofluorescence images of BODIPY were obtained after UA/Cis, UA/Cis/DFO, or UA/Cis/3-MA treatment (g, k) (*n* = 3, scale bar: 100 *μ*m). Intracellular Fe^2+^ levels were measured after treatment with UA/Cis, UA/Cis/DFO, or UA/Cis/3-MA (h, l) (*n* = 3, scale bar: 25 *μ*m). Measurement of TFR level by immunofluorescence staining of HOS cells after treatment with UA/Cis, UA/Cis/DFO, or UA/Cis/3-MA (i, m) (*n* = 3, scale bar: 50 *μ*m). The expression of iron metabolism-related proteins in UA/Cis-, UA/Cis/DFO-, or UA/Cis/3-MA-treated HOS cells was determined by western blotting (j) (*n* = 3). The data are presented as the mean ± SD. ^∗^*p* < 0.05 vs. the UA/Cis group.

Due to the significant increase in intracellular free iron levels and the significant activation of autophagy, we proposed that the synergistic toxic effect of UA and Cis on OS cells may be caused by the accumulation of free iron due to excessive autophagy. We tested the above hypothesis using the iron-chelating agent DFO and the autophagy inhibitor 3-MA. The evidence showed that DFO and 3-MA reduced UA+Cis-induced apoptosis (Figures 6(a) and 6(b)) and that DFO and 3-MA partially reversed the UA+Cis-induced decrease in cell viability (Figure 6(c)). In addition, UA+Cis-induced mitochondrial morphological changes and autophagy were partially reversed by DFO and 3-MA (Figure 6(d)). Furthermore, DFO and 3-MA were found to reduce lipid peroxidation and MDA and 4-HNE contents in OS cells (Figures 6(e)–6(g) and 6(k)). Notably, we demonstrated that DFO and 3-MA effectively alleviated the intracellular overload of free iron (Figures 6(h) and 6(l)). DFO and 3-MA also reduced the increased expression of TFR induced by UA+Cis treatment (Figures 6(i) and 6(m)). The western blot results showed that the expression levels of TFR, NCOA4, *γ*-H2AX, and LC3 were decreased while GPX4 and P62 expression was increased after treatment with the iron-chelating agent DFO and the autophagy inhibitor 3-MA (Figure 6(j)). These data suggested that the enhancement of autophagy induced by UA+Cis treatment led to the accumulation of free iron and that free iron overload acted upstream of mitochondrial dysfunction, ultimately leading to the production of lipid peroxides that cause ferroptosis. Taken together, these data indicated that pharmacological chelation of free iron and inhibition of autophagy effectively alleviated UA+Cis-mediated ferroptosis and mitochondrial dysfunction.

### 3.7. UA and Cis Treatment Increases Autophagy and Thus Induces Ferroptosis in Mouse Xenograft Models

**Figure 7:**
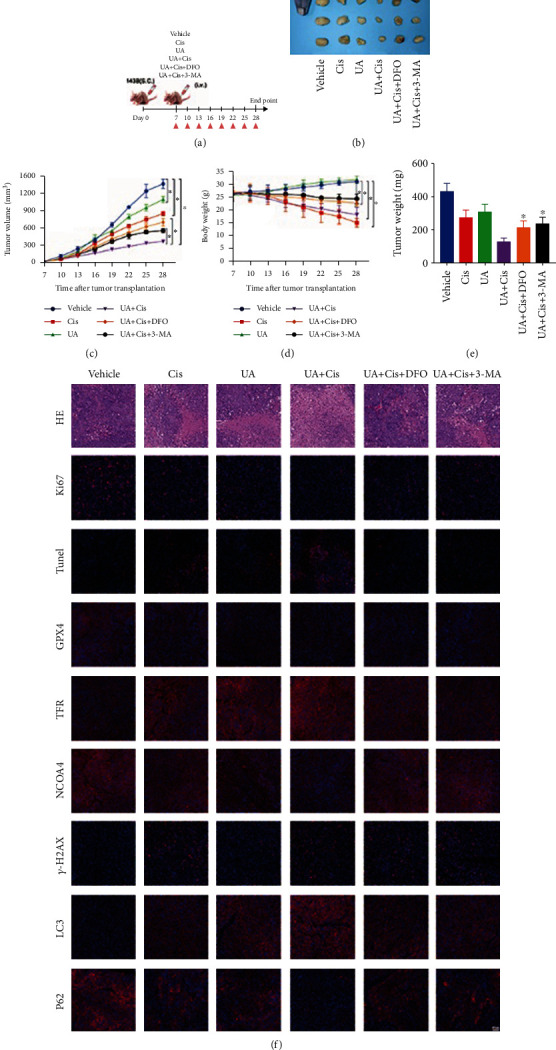
The combination of UA and Cis inhibited the growth of xenografts in vivo. Time flow chart of in vivo mouse xenograft models (a). Macroscopic view of xenograft tumours at the endpoint of the experiment (b) (*n* = 3). The average tumour volume and weight and body weight of the different groups (c–e) (*n* = 3). H&E staining and immunofluorescence staining of autophagy-, ferroptosis-, and iron metabolism-related proteins in xenograft tissue sections (f) (*n* = 3, scale bar: 50 *μ*m). The data are presented as the mean ± SD. ^∗^*p* < 0.05 vs. the control group.

We established a subcutaneous tumour model to study the therapeutic potential of the combination of UA and Cis in vivo, and tumour growth was observed periodically (Figure 7(a)). We found that compared with control treatment and monotherapy, UA+Cis treatment significantly slowed tumour growth (Figure 7(b)); the tumour volume and weight were significantly decreased in the UA+Cis treatment group compared with the control group and the monotherapy group (Figures 7(c) and 7(e)). As shown in Figure 7(d), the Cis-treated mice showed significant weight loss, while the weight of the UA-treated mice was almost unchanged, indicating no significant toxicity. It is worth noting that the combination of Cis and UA not only inhibited tumour growth but also reduced the toxicity and side effects of Cis to a certain extent, making it safer for clinical use. The immunofluorescence results showed that UA synergized with Cis to increase the necrotic area; increase the expression of TFR, *γ*-H2AX, and LC3; and decrease TUNEL, Ki67, GPX4, NCOA4, and P62 staining (Figure 7(f)). However, DFO and 3-MA significantly reversed these changes by inhibiting ferroptosis and autophagy. The results also confirmed that the combination of UA and Cis exerted a cytotoxic effect on OS cells by promoting autophagy, leading to ferroptosis due to intracellular overload of iron ions.

## 4. Discussion

Traditional treatments for OS mainly include a combination of surgery, chemotherapy, and radiation, as well as advanced alternative therapies such as targeted therapy and immunotherapy [13]. However, these therapies have limited efficacy, and their use is limited by their side effects and ultimately the development of multidrug resistance (MDR). Therefore, it is imperative to optimize cancer treatment strategies. For complex diseases such as cancer, TCM compounds can affect multiple targets rather than a single target, with a few exceptions [14]. UA, a natural pentacyclic triterpenoid found in plants, plays an anticancer role by targeting multiple signalling pathways and is becoming a promising compound for cancer prevention and treatment [15]. Notably, we demonstrated that UA synergizes with Cis to activate autophagy, leading to degradation of ferritin and resulting in the catastrophic accumulation of free iron and lipid peroxidation and thus ferroptosis, which has an inhibitory effect on OS.

It has been reported that UA can inhibit tumour cell growth, promote tumour cell apoptosis, and inhibit the invasion and metastasis of tumour cells to exert an antitumour effect [9, 16]. However, the regulatory effect of UA on OS has rarely been studied. Studies have shown that UA and Cis exert synergistic antitumour effects by inducing apoptosis of tumour cells, including ovarian cancer, bladder cancer, and lung adenocarcinoma cells [17–19]. In addition, the combination of UA and Cis can significantly disrupt mitochondrial homeostasis and inhibit OS cell proliferation. Further experiments have shown that iron-chelating agents and autophagy inhibitors can block cell death. Importantly, the accumulation of lipid peroxides before OS cell death was detected by staining for MDA and 4-HNE or fluorescent staining with BODIPY C11, and the accumulation of lipid peroxides could be reversed by pharmacological chelation of free iron. The same phenomenon was observed in a tumour xenograft model. In summary, we found that UA can effectively optimize the anti-OS activity of Cis and reduce the toxicity and side effects of Cis, which involve multiple mechanisms. UA not only increases DNA damage but also causes lipid peroxidation in OS cells, ultimately leading to ferroptosis. These results indicate that the application of UA combined with Cis is a promising strategy to decrease iron levels in OS.

Iron plays an important role in mammalian cells. Iron is necessary for cell replication, metabolism, and growth, as it allows important iron-containing enzymes in the blood to function [20]. Unstable iron pool overload is a biochemical hazard. Ferrous iron contributes electrons when it reacts with hydrogen peroxide, producing hydroxyl radicals, which are ROS [21, 22]. This process, known as the Fenton reaction, not only destroys lipids and proteins but also leads to oxidative damage to DNA, including modification of DNA bases and breakage of DNA strands [23]. Therefore, iron is both essential and potentially toxic. Ferritin, which includes ferritin light peptide 1 (FTL1) and ferritin heavy peptide 1 (FTH1), is the main intracellular iron-storage protein complex. Ferroptosis can be inhibited by increasing iron storage in inert pools through upregulation of cytoplasmic ferritin expression [24]. Autophagy is an evolutionarily conserved degradation mechanism that maintains homeostasis. Induction of autophagy promotes cell survival in response to environmental stresses such as nutritional starvation and energy expenditure in many cases. However, excessive and impaired autophagy may lead to cell death [25]. Recent studies have shown that increased autophagy can degrade ferritin and increase iron levels, leading to the Fenton reaction and oxidative damage [24]. Increasing evidence suggests that induction of ferroptosis is a promising therapeutic strategy for inhibiting cancer cell resistance resulting from drug-induced apoptosis [26]. Mechanistically, the combination of UA and Cis activates autophagy to degrade the iron storage protein ferritin through a process mediated by the cargo receptor NCOA4. This NCOA4-mediated autophagic degradation of ferritin, also known as ferritinophagy, promotes the accumulation of ROS by maintaining high intracellular iron levels, thus leading to ferroptosis of OS cells. In this paper, we present convincing evidence that ferroptosis is a form of autophagic cell death that causes OS cell death after UA and Cis combination therapy. It was noted that treatment with UA and Cis exacerbated DNA damage in OS cells and arrested OS cells in G2 phase of the cell cycle. These results suggest that the treatment of OS cells with the combination of UA and Cis can alleviate drug resistance by enhancing DNA damage and ferroptosis in a multistep and multimechanism manner.

Thus, our study provides evidence that UA may be a promising adjuvant for improving the effectiveness of Cis-based chemotherapy in patients with OS. We also reveal the underlying mechanisms to further elucidate the effect of targeting ferroptosis for OS therapy.

## 5. Conclusion

This study provides strong evidence that UA can strongly enhance the DNA damage induced by Cis. Moreover, UA and Cis can synergistically exert regulatory effects at multiple levels by affecting multiple targets simultaneously, making OS cells susceptible to ferroptosis. Notably, the properties of UA enhance its anticancer activity and its effect against chemotherapy resistance in OS, which may be a promising therapeutic strategy. This supports its use as an adjuvant to synergistically improve the efficacy of current chemotherapy regimens for OS.

## Data Availability

The raw data supporting the conclusions of this article will be made available by the authors, without undue reservation, to any qualified researcher.

## Abbreviations

OS: Osteosarcoma
Cis: Cisplatin
TCM: Traditional Chinese medicine
UA: Ursolic acid
DFO: Desferrioxamine
3-MA: 3-Methyladenine
CCK-8: Cell Counting Kit-8
EdU: 5-Ethynyl-2′-deoxyuridine assay
PI: Propidium iodide
ROS: Reactive oxygen species
MDA: Malondialdehyde
4-HNE: 4-Hydroxynonenal
H&E: Haematoxylin-eosin
MDR: Multidrug resistance
FTL1: Ferritin light peptide 1
FTH1: Ferritin heavy peptide 1
DCF-DA: 2′,7′-Dichlorofluorescein diacetate
TFR1: Transferrin receptor 1
NCOA4: Nuclear receptor coactivator 4.

## References

[1] Li F., Miao L., Xue T. Inhibiting PAD2 enhances the anti-tumor effect of docetaxel in tamoxifen-resistant breast cancer cells. *Journal of Experimental & Clinical Cancer Research*.

[2] Huang K., Chen Y., Zhang R. Honokiol induces apoptosis and autophagy via the ROS/ERK1/2 signaling pathway in human osteosarcoma cells in vitro and in vivo. *Cell Death & Disease*.

[3] Yen J. H., Huang S. T., Huang H. S. HGK-sestrin 2 signaling-mediated autophagy contributes to antitumor efficacy of Tanshinone IIA in human osteosarcoma cells. *Cell Death & Disease*.

[4] Arnesano F., Natile G. (2018). Interference between copper transport systems and platinum drugs. *Seminars in Cancer Biology*.

[5] Shaw V., Srivastava S., Srivastava S. K. Repurposing antipsychotics of the diphenylbutylpiperidine class for cancer therapy. *Seminars in Cancer Biology*.

[6] Ghoneum A., Almousa S., Warren B. (2021). Exploring the clinical value of tumor microenvironment in platinum-resistant ovarian cancer. *Seminars in Cancer Biology*.

[7] Chan D. W., Yung M. M., Chan Y. S. (2020). MAP30 protein from _Momordica charantia_ is therapeutic and has synergic activity with cisplatin against ovarian cancer _in vivo_ by altering metabolism and inducing ferroptosis. *Pharmacological Research*.

[8] Chen J., Fu H., Wang Z. (2014). A new synthetic ursolic acid derivative IUA with anti-tumor efficacy against osteosarcoma cells via inhibition of JNK signaling pathway. *Cellular Physiology and Biochemistry*.

[9] Pei Y., Zhang Y., Zheng K. Ursolic acid suppresses the biological function of osteosarcoma cells. *Oncology Letters*.

[10] Zhang R. X., Li Y., Tian D. D. Ursolic acid inhibits proliferation and induces apoptosis by inactivating Wnt/*β*-catenin signaling in human osteosarcoma cells. *International Journal of Oncology*.

[11] Li T., Yin Y., Mu N. Metformin-enhanced cardiac AMP-activated protein kinase/atrogin-1 pathways inhibit charged multivesicular body protein 2B accumulation in ischemia-reperfusion injury. *Frontiers in Cell and Development Biology*.

[12] Rottenberg S., Disler C., Perego P. The rediscovery of platinum-based cancer therapy. *Nature Reviews. Cancer*.

[13] Ding Q., Zhang W., Cheng C. Dioscin inhibits the growth of human osteosarcoma by inducing G2/M-phase arrest, apoptosis, and GSDME-dependent cell death in vitro and in vivo. *Journal of Cellular Physiology*.

[14] Yao C. L., Zhang J. Q., Li J. Y., Wei W. L., Wu S. F., Guo D. A. (2020). Traditional Chinese medicine (TCM) as a source of new anticancer drugs. *Natural Product Reports*.

[15] Yarla N. S., Bishayee A., Sethi G. Targeting arachidonic acid pathway by natural products for cancer prevention and therapy. *Seminars in Cancer Biology*.

[16] Sommerwerk S., Heller L., Kuhfs J., Csuk R. Urea derivates of ursolic, oleanolic and maslinic acid induce apoptosis and are selective cytotoxic for several human tumor cell lines. *European Journal of Medicinal Chemistry*.

[17] Lin K. W., Huang A. M., Lin C. C. Anti-cancer effects of ursane triterpenoid as a single agent and in combination with cisplatin in bladder cancer. *European Journal of Pharmacology*.

[18] Wang Y., Luo Z., Zhou D. Nano-assembly of ursolic acid with platinum prodrug overcomes multiple deactivation pathways in platinum-resistant ovarian cancer. *Biomaterials Science*.

[19] Li Y., Xing D., Chen Q., Chen W. R. Enhancement of chemotherapeutic agent-induced apoptosis by inhibition of NF-kappaB using ursolic acid. *International Journal of Cancer*.

[20] Torti S. V., Torti F. M. Iron and cancer: more ore to be mined. *Nature Reviews. Cancer*.

[21] Salnikow K. (2013). *Role of iron in cancer*.

[22] Garon E. B., Brodrick P. Targeted therapy approaches for MET abnormalities in non-small cell lung cancer. *Drugs*.

[23] Bordini J., Morisi F., Elia A. R. Iron induces cell death and strengthens the efficacy of antiandrogen therapy in prostate cancer models. *Clinical Cancer Research*.

[24] Tang M., Chen Z., Wu D., Chen L. Ferritinophagy/ferroptosis: iron-related newcomers in human diseases. *Journal of Cellular Physiology*.

[25] Hou W., Xie Y., Song X. Autophagy promotes ferroptosis by degradation of ferritin. *Autophagy*.

[26] Greco G., Catanzaro E., Fimognari C. Natural products as inducers of non-canonical cell death: a weapon against cancer. *Cancers (Basel)*.

